# Optimization of Dominant Frequency and Bandwidth Analysis in Multi-Frequency 3D GPR Signals to Identify Contaminated Areas

**DOI:** 10.3390/s22249851

**Published:** 2022-12-15

**Authors:** David Paredes-Palacios, Francisco Mota-Toledo, Bárbara Biosca, Lucía Arévalo-Lomas, Jesús Díaz-Curiel

**Affiliations:** 1 Department of Geological and Mining Engineering, School of Mines and Energy, Universidad Politécnica de Madrid, C/Ríos Rosas 21, 28003 Madrid, Spain; 2 Geofísica Aplicada Consultores, S.L., C/José Luis Velasco 3, 28250 Torrelodones, Spain; 3 Department of Energy and Fuels, School of Mines and Energy, Universidad Politécnica de Madrid, C/Ríos Rosas 21, 28003 Madrid, Spain

**Keywords:** ground-penetrating radar (GPR), frequency domain, bandwidth, dominant frequency, resistivity, LNAPL

## Abstract

Ground-penetrating radar (GPR) has been widely used in investigations of contaminated areas because of its sensitivity to variations associated with the nature of pore fluids. However, most of the studies were usually based on the visual interpretation of radargrams or on a time domain amplitude analysis. In this work, we propose a methodology that consists of analyzing the spectral content of the signal recorded in multi-frequency 3D GPR profiles. A remarkable advantage of this type of antenna is its step-frequency system, which provides a much wider emission spectrum than the one corresponding to conventional single-frequency antennas. From the data in the frequency domain, the dominant frequency and bandwidth were calculated as parameters whose variation could be related to the presence of light non-aqueous phase liquid (LNAPL) in the subsurface. By analyzing the variations of these two parameters simultaneously, we were able to delimit the contaminated zones in a case study, associating them with a significant shift of the frequency spectrum with respect to the average of the study area. Finally, as a validation method of the proposed methodology, the results of the frequency analysis were compared with resistivity data obtained with an electromagnetic conductivity meter, showing a very good correlation between the results.

## 1. Introduction

The peculiarities of ground-penetrating radar (GPR) surveying enable the characterization of certain parameters with a degree of resolution and speed of data acquisition that exceeds the possibilities offered by other current geophysical methods [1,2]. However, the visual interpretation of GPR recordings is often highly subjective, making it necessary to introduce tools that allow quantitative analysis of the signals and eliminate interpretive uncertainties [3,4].

In this regard, one of the procedures that allows this process to be carried out is the spectral analysis of the GPR reflections [5,6,7,8]. This spectral content is largely influenced by the electromagnetic properties of the materials of the analyzed medium, causing a variation in frequencies along the traces, resulting in a spectral shift towards frequencies different from those emitted [9]. This shift is particularly pronounced in materials with high attenuation rates [10,11]. Thus, in a mainly sandy soil, the spectral content of the GPR signal is much richer than that in a predominantly clayey medium. Similarly, moisture or the presence of certain contaminants can strongly attenuate the high frequencies, limiting the frequency range of the recorded signal [12,13]. Therefore, by analyzing the frequency variations as a time function, some information regarding the electromagnetic properties of the materials under investigation can be obtained [14,15,16].

Focusing on the frequency, some of the attributes related to the frequency spectrum that can be obtained for different time windows are as follows: the dominant frequency (frequency with the highest amplitude), the maximum spectral amplitude (amplitude of the dominant frequency), the average frequency (arithmetic mean of the frequency spectrum), the bandwidth (width of the frequency spectrum for a specified amplitude), and the frequency differential (positive or negative difference between the amplitude spectrum of each trace with respect to the average spectrum of the profile) [17].

Considering all the above, in the present work, two of these parameters (dominant frequency and bandwidth) were identified using the spectral analysis and the attributes of the recorded signal, analyzing each trace separately. Therefore, with the proposed methodology we try to perform a different treatment of the GPR signal. For this purpose, the GPR data were transformed to the frequency domain and the dominant frequency and bandwidth have been analyzed, with the aim to relate the variation of these two parameters with the location of possible contaminated areas.

Complementarily, the data obtained after the application of this methodology were correlated with resistivity values derived from the application of other electromagnetic methods.

### Background

Soil contaminated by hydrocarbons is a problem that affects soil and water quality in both urban and industrial areas. Contaminants in the subsoil can exist as a separate, immiscible liquid phase, known as non-aqueous phase liquid (NAPL). NAPLs basically consist of a solution of organic compounds that can be either light (LNAPL) or dense (DNAPL). LNAPLs (e.g., petroleum hydrocarbons) form a layer that is less dense than water and floats above the water table [18,19].

GPR has been widely used to study shallow structures because of its high resolution and its sensitivity to variations associated with the nature of pore fluids [20]. The GPR method is a non-invasive geophysical technique based on variations in the electromagnetic properties of materials present in the subsurface [21]. A transmitting antenna emits a periodic short-duration pulse that, after being reflected in the possible heterogeneities of the medium, is recorded by the receiving antenna [22].

The importance of being able to characterize the subsurface and locate this type of contamination has led many researchers in recent years to study the application of GPR in the detection of LNAPL in the subsurface. These studies were mostly based on the visual interpretation of radargrams [23,24,25] or on a time-domain analysis of amplitudes [26,27,28]. However, the main inconvenience of these methods is that certain hydrogeological or stratigraphic changes may generate anomalies that are difficult to distinguish from LNAPLs [29]. For this reason, in order to apply GPR to the identification of an area contaminated by LNAPLs, it is necessary to have a complementary method to differentiate these types of anomalies. GPR and electrical methods have been applied in the study of contaminated areas, obtaining satisfactory results based on the analysis of the electromagnetic properties (permittivity and resistivity) of the subsoil [30,31,32].

Generally, in the frequency range corresponding to the GPR, the conductivity of LNAPLs is usually lower than that of groundwater; however, certain processes, such as bacterial biodegradation of some hydrocarbons in the dissolved phase, can cause a contaminated area to have notably high conductivities [33]. The conductivity of the medium is directly related to the attenuation of GPR waves and their spectral content [11,34]. Therefore, by analyzing the morphology of the signal in the frequency spectrum, it is possible to obtain information about the location of possible contaminants in the subsoil of the investigated area.

In recent decades, several authors studied some of the parameters that can be obtained from the frequency spectrum of the GPR signal [35,36,37]. In this work, we propose a methodology based on the joint analysis of two parameters (dominant frequency and bandwidth) that are related to the GPR signal in the frequency domain and the results obtained are compared with resistivity values measured in the same study area.

## 2. Methodology and Instrumentation

The dominant frequency and bandwidth, determined in the frequency domain, are related to the moisture content or to the presence of certain contaminants in the investigated medium [38,39]. The difference in the electromagnetic properties of the investigated media will cause, according to the theory of wave propagation, a change in the reflected waves. Thus, the signal spectrum of the reflected waves presents different frequencies (higher or lower depending on the medium), so is to be expected that the bandwidth and the dominant frequency are modified in the presence of contamination.

Therefore, the first step of the process consists of transforming each GPR trace and obtaining its frequency spectrum by applying the fast Fourier transform (FFT). Afterwards, from the data in the frequency domain, the dominant frequency and bandwidth are obtained as indicative parameters, the joint analysis of which allows the identification of the areas of subsurface contamination.

### 2.1. Analysis of Attributes in the Frequency Domain

A radargram is comprised of a succession of traces, each of which constitutes the signal (reflections of the emitted electromagnetic wave) recorded by the GPR antenna in the time domain [40]. To study the information contained in each trace, from a frequency analysis, it is necessary to transform the trace in the time domain into the frequency domain [41]:

(1) $$x\left(t\right)\to X\left(f\right)=\overset{\infty }{\underset{-\infty }{\int }}x\left(t\right)\cdot e^{-i2\pi tf}dt,$$

where x(t) and X(f) are the signals in the time and frequency domains, respectively.

Using the fast Fourier transform (FFT) as a quick procedure to calculate the discrete Fourier transform (DFT), the number of operations to be performed is considerably reduced, moving from the order of $N^{2}$ operations to the order of N·log(N), N being the dimension of the input vector. One of the most widely used algorithms for FFT implementation is the Cooley–Tukey algorithm [42]. This is a radix-2 algorithm, which, at each step of the recursive process, divides the problem into two subproblems of smaller size than the original problem.

We define the discrete Fourier transform for $x_{0},\;x_{1},\;\ldots ,\;x_{N-1}$ as:

(2) $$X_{k}=\overset{N-1}{\underset{n=0}{\sum }}x_{n}e^{-\frac{2\pi i}{N}nk},$$

where N is the total number of samples for the input signal and k is the frequency analyzed in each iteration [43]. The part corresponding to the complex exponential $\left(e^{-\frac{2\pi i}{N}nk}\right)$ is referred to as the twiddle factor.

The Cooley–Tukey algorithm is based on restructuring the original N sample signal to $N=N_{1}\cdot N_{2}$. For this new data matrix, the DFT is calculated for each of its columns ($N_{1}$) by sweeping all frequencies. Next, each of the obtained factors is multiplied by the twiddle factor and the DFT is calculated for each row ($N_{2}$). Finally, the data are rearranged into a vector of N samples, as in the original format [44,45].

Everything described above can be implemented using a programming language, with some precautions. First of all, according to the sampling theorem, the minimum number of samples needed to avoid aliasing effects must be at least twice the maximum frequency, which is the so-called Nyquist frequency, and its size must be equal to a power of 2 [46]. Second, when applying the FFT to a signal, bilateral amplitude and phase spectra are obtained. The bilateral amplitude spectra are divided into positive and negative frequencies, in an even symmetry, and the amplitudes obtained are those corresponding to ½ of the original signal.

Once the transformation of each trace to the frequency domain is obtained, the analysis of some of their attributes can begin.

**Figure 1:**
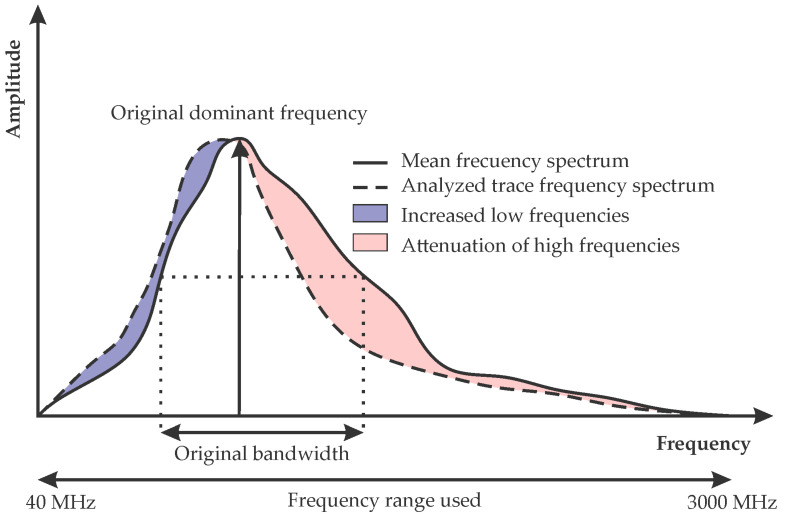
Diagram of the typical frequency spectrum in GPR and of the two parameters analyzed in this study.

First, the value of the dominant frequency for each trace is determined. This value is defined as the frequency corresponding to the maximum amplitude of each trace in the frequency spectrum [40]. Second, the bandwidth is calculated as the frequency range in which the amplitudes, of the frequency spectrum, are above a specified limit (Figure 1). This limit for the calculation of the bandwidth is determined from the average frequency spectrum for the entire study area and should be analyzed for each case study.

Through the simultaneous analysis of these two parameters, it is possible to determine the areas in which there is a notable variation with respect to the average frequency spectrum of the GPR profile, produced by the presence of certain contaminants.

### 2.2. Multi-Frequency 3D GPR

It is important to note that the methodology proposed in this work is applicable to any type of GPR antenna and to any emission frequency, as long as it is adapted to the peculiarities of the study and the investigated medium.

**Figure 2:**
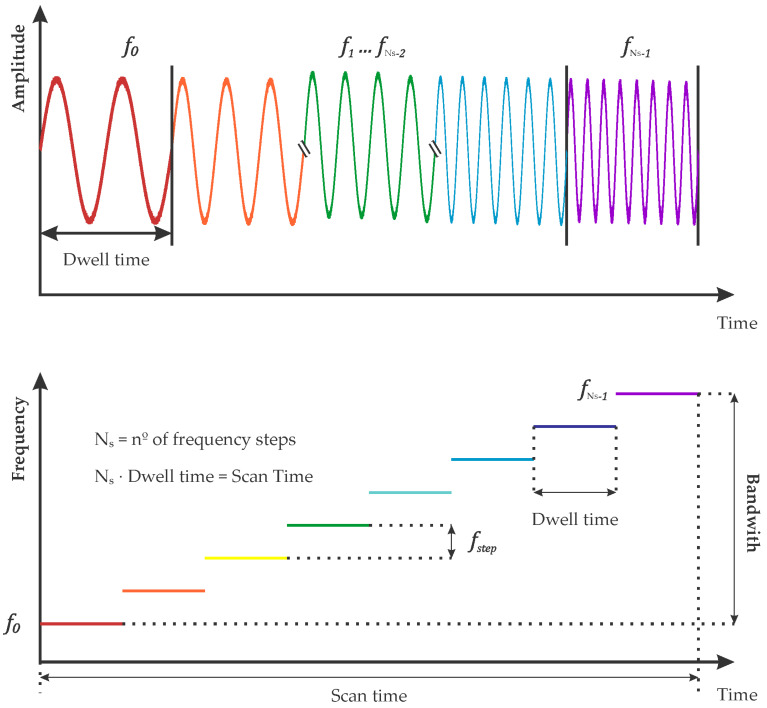
Descriptive diagram of the step-frequency system and all its associated parameters.

The antenna used for the acquisition of GPR data in this study was a 3D multi-frequency antenna of the Norwegian company Kontour. This type of antenna uses a step-frequency scanning system (Figure 2), in which the antenna is capable of emitting multiple pulses at specific frequencies ranging from 40 MHz to 3000 MHz, obtaining a wider frequency spectrum than that of conventional single-frequency antennas [47].

This type of GPR antenna emits consecutive streams of sinusoidal signals at different frequencies, receives reflected signals for each stepped frequency, and converts them to an intermediate frequency (IF) signal, which is then demodulated into baseband in-phase (I) and quadrature (Q) signals. Amplitude and phase information, real and imaginary parts of the received signal, are contained in I and Q, respectively. Using an analogical–digital converter, these signals are transformed into a digital synthetic pulse in the time domain by applying inverse Fourier transform [48]. The transmitted frequencies $\left(f_{0},\;f_{1},\;\ldots ,\;f_{Ns-1}\right)$ are separated with a uniform frequency increment (step-frequency). The total bandwidth is obtained as:

(3) $$B=f_{Max}-f_{min}=N_{s}\cdot f_{step},$$

where B is the total bandwidth, $N_{s}$ is the number of frequency steps, and $f_{step}$ is the step frequency. The last parameter describing the GPR signal in the step-frequency system is the dwell time or pulse repetition interval, which is defined as the time taken to transmit the signal corresponding to each frequency [49].

## 3. Case Study and Location

### 3.1. Location and Geology

The data used in the present study were acquired from an urban area located in the east of the province of Madrid (Spain). Geologically, the area is located within the meso-tertiary Tajo Basin or Madrid Basin in the transition zone of the intermediate to central facies of the basin [50].

**Figure 3:**
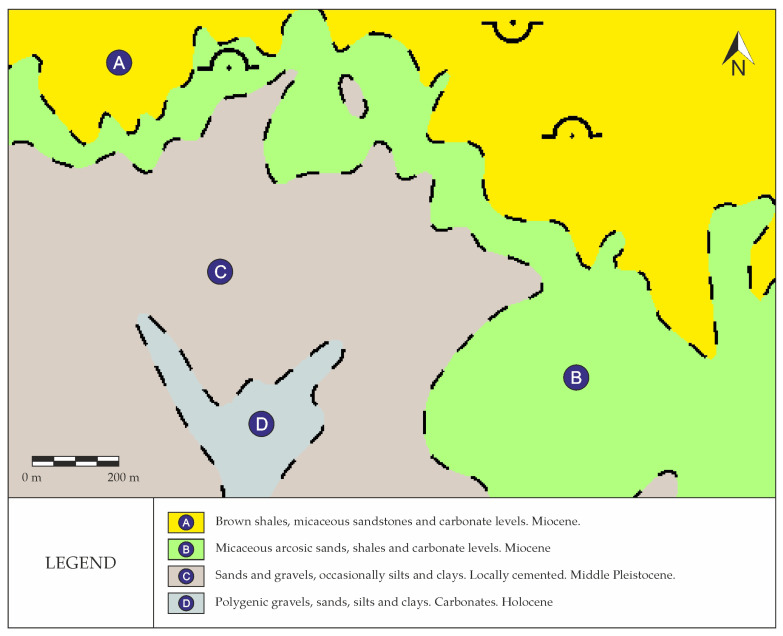
Digital continuous geological map at a scale of 1:50,000, zone 2400 (Tajo-Mancha Basin).

Based on the available geological information (©Instituto Geológico y Minero de España), obtained from the digital continuous geological ap at a scale of 1:50,000, zone 2400 (Tajo-Mancha Basin), the materials present in the study area and its surroundings consist mainly of Cenozoic detritic materials together with Quaternary fluvial deposits (Figure 3).

From the general geomorphologic point of view, the reliefs of the Henares and Jarama Rivers in terraces stand out [50].

Regarding the hydrogeological characteristics of the area, the complex of Miocene detritic materials can be considered as a single aquifer, with diverse heterogeneities originating in a system of fluvial fans. The gravel and sand levels originated from the major river channels, the sandy clays and clay sands derived from inundation deposits, whereas the clayey deposits correlated with mudflows generated in the sedimentary environment. As for the Quaternary fluvial terraces, they can be considered as aquifers with primary permeability owing to intergranular porosity, whose recharge comes both from precipitation and from the discharge of the Tertiary aquifer in the valleys [50].

### 3.2. Case Study and Data Acquisition

The present study was conducted in an urban area through which a metallic pipeline for transporting hydrocarbons (mainly diesel for domestic use) runs. This pipe, with a diameter of 30 mm, is located at an average depth of about 0.3 m and, according to the information initially provided, presents a possible leak that has led to subsoil contamination in an undetermined extension.

**Figure 4:**
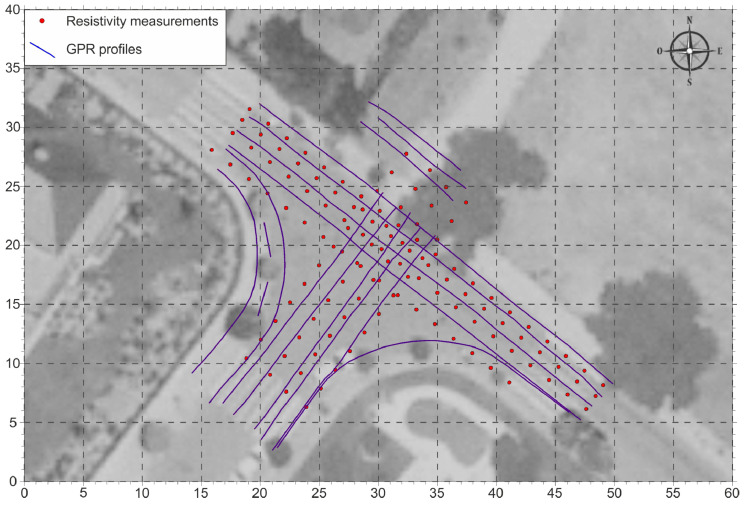
Location of the GPR profiles (blue lines) and resistivity measurements (red dots) of the study area.

To attempt to delimit the area affected by the possible leakage, a combined geophysical investigation was developed, based on GPR profiles and resistivity measurements made with an electromagnetic conductivity meter, distributed as shown in the map of Figure 4.

The data corresponding to the multi-frequency 3D GPR were acquired over a total of 17 profiles, using a ground-coupled antenna array of 12 channels for a total of 376.2 linear meters analyzed for each channel. This study was carried out using the 12 available channels, performing a standard GPR test, thus, these 12 profiles were obtained with a lateral separation of 7.5 cm for each sweep of the antenna.

For the effective movement of the equipment, an auscultation cart was used, on which the GPR antenna, the control unit, the display and recording computer, the GPS equipment, and the power supply batteries were attached.

The GPR pulses were configured to cover a bandwidth from 40 MHz to 2990 MHz, using a step frequency of 10 MHz. This implies that each recorded trace was obtained from the reflections generated by a total of 296 pulses of different frequencies for each of the 12 channels used.

The longitudinal separation between traces (trigger interval) was 0.94 cm, which was measured using an odometer wheel specifically adapted to the auscultation cart. The position for each trace was georeferenced with the odometer records combined with the data acquired using a centimeter-accurate GPS.

With the electromagnetic conductivity meter, a total of eight profiles were made with discrete measurements every 2 m, applying a measurement time of 2 s for each point. The equipment used operates with a maximum sampling rate of 10 kHz and was configured for the acquisition of apparent resistivity (inverse of conductivity) measurements of the ground at depths of 1.1, 2.1, and 3.3 m. Each measurement station was geo-referenced using a GPS synchronized with the electromagnetic conductivity meter.

### 3.3. Data Processing

To correctly apply the methodology proposed in this study, the GPR data were preprocessed prior to the frequency analysis of the signal.

Once the zero-time correction was performed, the first step of signal preprocessing consisted of eliminating the DC-shift present in any GPR record. To avoid possible distortions in the later stages of analysis, it is important to eliminate this effect in the first processing step [51,52,53]. For this purpose, the median of all amplitude values after the initial strong undulations caused by the first reflection was calculated for each trace [54]. The obtained value was subtracted from that of each of the trace sample. Thus, the entire trace shifted to its correct reference position without affecting its amplitude values or frequency content. The use of the median is a criterion shared by several authors [55,56] to avoid the influence of extreme values.

The second step of the signal preprocessing consisted of compensating the exponential decay of the signal, using the logarithmic transformation of the amplitudes [57]. In this way, the energy loss of the amplitudes corresponding to the last arrival times was compensated.

Subsequently, the previously preprocessed signal was transformed to the frequency domain by applying the FFT, as indicated in Section 2.1 of this study, and the two parameters (dominant frequency and bandwidth) on which our spectral analysis is based are calculated.

## 4. Results

In general, the presence of LNAPL in the subsurface significantly increases the resistivity, but regarding the dissolved phase, several authors reported some mechanisms, such as the dissolution of salts or the production of organic and carbonic acids during biodegradation, which can lead to elevated conductivities compared with those the surrounding clean zones [58,59,60,61,62,63,64].

Based on these previous studies and the available information on the approximate location of the contamination plume, in this work, we associated the presence of contaminants with an increment in the conductivity and attenuation of the GPR signal. This is represented by a shift in the frequency spectrum towards lower values than those corresponding to LNAPL-free zones.

**Figure 5:**
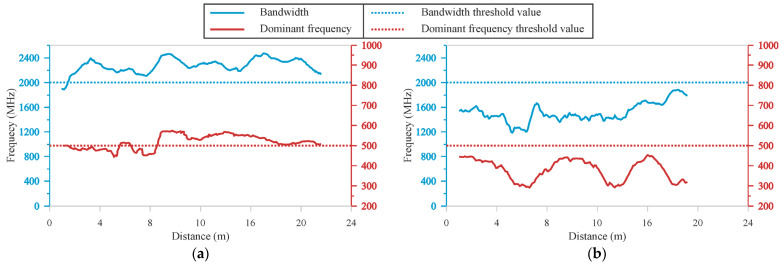
Examples of the results obtained for an area free of contaminant (**a**) and with the presence of LNAP (**b**).

After obtaining the dominant frequency and bandwidth values for each trace, joint plots of the two values were generated for each GPR profile (Figure 5). In this study, using a comparative analysis of uncontaminated and potentially contaminated areas, a threshold of 500 MHz for the dominant frequency and 2000 MHz for the bandwidth, calculated for an amplitude of 0.5 in normalized values, was determined. This means that when the dominant frequency and bandwidth values are below the established threshold, it can be associated with the presence of LNAPL.

**Figure 6:**
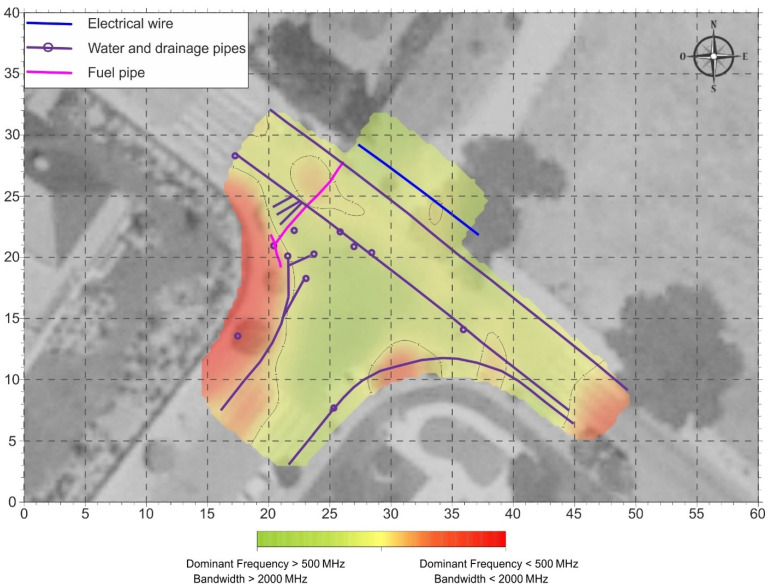
Distribution map of the results obtained from the dominant frequency and bandwidth analysis. The color scale shows a gradient from green to red according to the limits established for the two parameters analyzed in this study.

From the results obtained throughout the study area, it was possible to draw up a surface distribution map of the joint dominant frequency and bandwidth values (Figure 6). This map shows, in red colors, those areas in which the values obtained for the dominant frequency and bandwidth are below the threshold established in this study, and can, therefore, be associated with areas with LNAPL.

For this research, the variations of the frequency spectrum were studied using a time window of 100 ns. Analyzing the GPR signal in the frequency domain, the information concerning the investigation depth is not available, so it is only possible to determine the surface distribution of the identified anomalies.

### Correlation with Electrical Resistivity Values

In order to evaluate the validity of the proposed methodology, the results obtained from the analysis of the GPR data were compared with the resistivity values determined by measurements made in the same area, using an electromagnetic conductivity meter.

**Figure 7:**
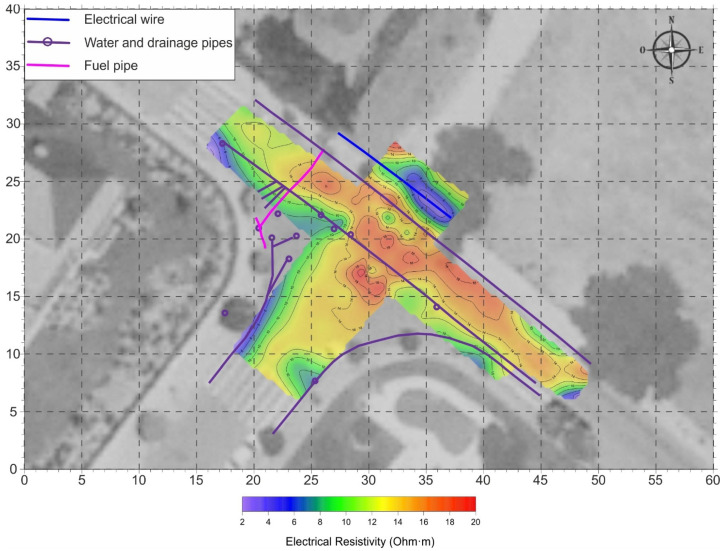
Resistivity distribution map obtained from electromagnetic conductivity measurements. The values represented correspond to 1.1 m depth.

The low resistivity values obtained (Figure 7), corresponding to a depth of 1.1 m, could be associated with silty or silty clay materials and the resistivity distribution shows that there is a very good correlation in the delimitation of the contaminated zone when applying the two techniques. Therefore, we can affirm that the methodology proposed in this study is applicable to the identification of hydrocarbon-contaminated zones.

As can be observed, in the resistivity measurements there is a conductive anomaly located to the NE of the figure, which is associated with the presence of a subterranean electrical conduction. This type of anomaly can be easily identified in a previous analysis of the GPR data, which were used to generate a distribution plan of buried services present in the study area.

## 5. Discussion and Conclusions

GPR prospecting is a geophysical technique applied in many different fields of engineering, geology, and environmental studies, but its most widespread method of use is based on the visual identification of layers and reflectors. This treatment of GPR data has a significant drawback in that it is conditioned to subjective interpretation of the results. For this reason, we presented here a methodology that allows researchers to treat the information provided by the GPR in a quantitative way by analyzing the spectral content of the data.

In this study, we transformed the GPR data to the frequency domain and analyzed the dominant frequency and bandwidth of each trace as parameters that can be related to the presence or absence of LNAPL in the subsurface. By analyzing these two parameters jointly, we are able to delimit the contaminated zones, associating them with those in which there is a notable variation with respect to the average frequency spectrum of uncontaminated study areas. Therefore, by applying the proposed methodology, we can quantitatively analyze the GPR signal and extract information from the radargrams that would be impossible to obtain by performing only a conventional visual analysis.

The emission bandwidth is an important factor in the application of this methodology. Conventional single-frequency antennas have a lower bandwidth, therefore, having a lower frequency content, so it is reasonable to assume that a certain resolution capacity will also be lost. However, although a multi-frequency 3D GPR antenna is used in this study, the proposed methodology is applicable to any type of GPR antenna, as long as the emission frequency used is adapted to the depth of investigation and the characteristics of the medium under analysis.

Although the presence of LNAPL is generally associated with high resistivity, different mechanisms can lead to an increase in conductivity in areas contaminated by certain hydrocarbons. In this study, we verify that the presence of LNAPL in the subsurface produces a shift in the frequency spectrum towards values lower than the average of the area, which can be associated with an increase in the conductivity of the medium and the attenuation of the GPR signal.

In the study area, resistivity measurements were carried out using an electromagnetic conductivity meter as a method to contrast the results obtained in this work. A very good correlation was obtained between the resistivity values and the results corresponding to the dominant frequency and bandwidth analysis.

As usual in the application of any geophysical method, the usefulness of the methodology proposed in this work is conditioned by the contrast between the frequency spectrum of each analyzed trace and the average of uncontaminated study areas. In such cases when the contrast is very low, it is not possible to accurately delimit the contaminated zones by analyzing the dominant frequency and bandwidth.

The methodology proposed in this study is not an attempt to replace the electrical or electromagnetic survey techniques, but to complement them. It is important to highlight that with the analysis of the GPR data in the frequency domain, we lose critical information regarding the depth of the anomalies identified. Nevertheless, this notable weakness of the data frequency analysis method constitutes the main challenge to be addressed in future studies, since the approximate depths of certain anomalies can be estimated by applying this methodology to different time windows along the acquired signal. Other possible lines of work that could be developed would consist of adding to the dominant frequency and bandwidth analysis other parameters referred to in the introduction, such as the frequency differential or the maximum spectral amplitude.

In conclusion, the methodology presented here allows for obtaining fast results related to the presence of LNAPLs in subsurface, which depends directly on the conductivity and attenuation of the medium under GPR investigation. In contrast, the electrical and electromagnetic methods used to obtain resistivity values have the usual operational drawbacks and slower data acquisition, especially in urban areas where GPR is generally much easier to implement.

## Data Availability

The data presented in this study are available on request from the corresponding author. The data are not publicly available due to some special reasons.
